# Exploring The Legal Landscape of Islamic Fintech in Indonesia: A Comprehensive Analysis of Policies and Regulations

**DOI:** 10.12688/f1000research.143476.2

**Published:** 2024-06-28

**Authors:** Dwi Fidhayanti, Mohd Shahid Mohd Noh, Ramadhita Ramadhita, Syabbul Bachri

**Affiliations:** 1 Islamic Economic Law, Universitas Islam Negeri Maulana Malik Ibrahim Malang, Malang, East Java, 65144, Indonesia; 2 Universiti Malaya, Kuala Lumpur, Malaysia; 3 Universitas Islam Maulana Malik Ibrahim Malang, Malang, Indonesia

**Keywords:** Indonesia policies, Indonesia regulations, Islamic Fintech

## Abstract

The rapid growth of Islamic fintech in Indonesia necessitates a comprehensive examination of the policy and regulatory framework from a legal perspective. This study explores the legal landscape of Islamic fintech in Indonesia, analyzing the current policies and regulations governing this emerging sector. Using normative legal research with a statutory approach and conceptual approach. The primary and secondary legal materials, including government reports and scholarly articles, this research evaluates recent policy developments and regulatory initiatives supporting Islamic fintech. It identifies gaps and areas for improvement, drawing from best practices and regulatory approaches adopted by other countries with successful Islamic fintech ecosystems. Based on the findings, this research proposes recommendations to enhance the policy and regulatory framework. These include collaboration between regulatory bodies and industry stakeholders, tailored licensing frameworks, enhanced consumer protection mechanisms, and promotion of innovation in Shariah-compliant financial products and services. This study contributes to understanding the legal landscape of Islamic fintech in Indonesia, offering a comprehensive analysis of existing policies and regulations. By addressing challenges and proposing solutions, it aims to facilitate the growth of Islamic fintech and foster an inclusive and sustainable financial ecosystem in Indonesia.

## Introduction

Islamic fintech has received significant attention ( Alshater et al., 2022; Dawood et al., 2022; Rabbani et al., 2021), especially in the Indonesia context ( Ascarya & Sakti, 2022; Hudaefi, 2020; Hudaefi et al., 2023; Muryanto et al., 2021). However, Islamic fintech in Indonesia is still in an early stage of development and is less comprehensive than conventional forms ( Aulia et al., 2020). There are many startups that do not comply with the Sharia principles that have been regulated by the Indonesian government ( Achsien & Purnamasari, 2016). To be fully sharia-compliant, these platforms must avoid interest-based transactions that are common in the fintech world. This restriction limits Islamic fintech companies’ products and services. There are several reasons for the slow growth of Islamic fintech in Indonesia, including inadequate regulations, complicated licensing procedures, rampant illegal fintech businesses, and consumer disputes in the fintech sector ( Muryanto et al., 2021). Nevertheless, Indonesia has huge potential for Islamic fintech. With the right policies and regulations, Islamic fintech could contribute to social funds and finance the agriculture sector, as well as small and micro businesses ( Agustina & Faizah, 2023). In addition, the adoption of fintech in Islamic finance assists the government in increasing financial inclusion, conquering financial crises such as COVID-19, and achieving the SDGs for a sustainable country ( Alshater et al., 2022). The rise of Islamic fintech in markets such as Indonesia and Malaysia is closely related to the growth of Islamic finance in Southeast Asia. According to the World Bank, Indonesia has the largest number of Islamic fintech companies in the world ( Hudaefi, 2020).

The development of fintech companies over time has been proven to improve banks’ financial stability of banks ( Safiullah & Paramati, 2022). A study was conducted to explore the evolving legal landscape surrounding Islamic fintech in Indonesia. Muryanto et al. (2021) found that Indonesia, despite having the largest Muslim population in the world, still lags behind Saudi Arabia, Iran, the United Arab Emirates, and Malaysia in terms of the Islamic fintech market size ( Muryanto et al., 2021). The Islamic fintech ecosystem consists of financial consumers, fintech startups, the government, technology developers, traditional financial institutions, and fatwa. Hudaefi examined the growth of the Islamic fintech ecosystem in Indonesia within these core elements and provided valuable insights into the current state of the industry in Indonesia ( Hudaefi et al., 2023). In Indonesia, Islamic fintech complies with *maqashid sharia* ( Nafiah & Faih, 2019). One of the points of Sharia compliance in Islamic fintech is the existence of the Sharia supervisory board to ensure compliance with Sharia provisions ( Iqbal, n.d.; Muryanto et al., 2021). The implementation of Islamic fintech in protecting the spiritual rights of customers by ensuring that Islamic fintech financing services do not involve interest or usury ( *riba*), uncertainty ( *gharar*), speculation ( *maysir*), hiding damage ( *tadlis*), harming other parties ( *dharar*), or avoiding things ( *haram*) ( Sugiarto & Disemadi, 2020). New regulations based on products, policies, and procedures are needed in order to gain more acceptance for the Islamic finance industry ( Mazlan et al., 2023). This research aims to examine the gap between the current and desired state of Islamic fintech regulations in Indonesia. By conducting a comprehensive analysis of policies and regulations, this research intends to provide insights to guide the development of a robust legal framework that matches the unique attributes of Islamic fintech. This article makes a significant contribution to the development of Islamic fintech in Indonesia by providing a comprehensive understanding of the legal landscape, addressing regulatory challenges, and providing actionable insights to create a conducive environment for sustainable growth and innovation in the sector.

## Method

The focus of this research is to explore comprehensively analyse the legal landscape of Islamic fintech in Indonesia. The research primarily employs a normative legal approach, aiming to examine and analyse existing policies and regulations related to Islamic fintech. To achieve this, both a statutory approach and a conceptual approach were utilized. The statutory approach involves an examination of government regulations, fatwa, and regulatory policies relevant to both law and fintech. Meanwhile, the conceptual approach is employed to delve into the principles of fintech and sharia that serve as references for ensuring sharia compliance within the Islamic fintech industry.

The data sources of this research encompass a variety of primary and secondary legal materials. The primary legal materials consist of legislative documents directly related to Islamic fintech in Indonesia. In addition, secondary legal materials such as national and international journal articles, theses, dissertations, and other scholarly works pertaining to the development of Islamic fintech regulations in Indonesia were gathered through a comprehensive literature study.

Interpretative analysis of the gathered data was conducted using both descriptive and comparative methods. The descriptive approach is employed in order to make comparisons with the countries that have a burgeoning Islamic fintech sector. The analysis results are then interpreted with a multifaceted lens, encompassing aspects of Sharia law, practical implications, and potential effects on the growth of the Islamic fintech industry in Indonesia. This interpretative phase adds depth and context to the findings, enabling a more comprehensive understanding of the legal framework surrounding Islamic fintech in the country.

### The Legal Landscape of Islamic Fintech in Indonesia

“FinTech” is a rather simple and obvious combination of an application domain (“financial”) and “technology” ( Alt et al., 2018). It refers to the integration of digital technologies into financial services, which has the potential to transform the provision of financial services and spur the development of new ones ( Feyen et al., 2023). The ongoing digitization of financial services and money creates opportunities to rapidly build a more inclusive and efficient financial sector landscape and blurs the boundaries of both financial firms and the financial sector, which presents a paradigm shift that has various policy implications ( World Bank, 2023). Some of these policy implications include fostering beneficial innovation and competition while managing risks, broadening monitoring horizons and reassessing regulatory perimeters, ensuring that public money remains fit for the digital world, and pursuing strong cross-border coordination. Digital transformation has become a disruptor in almost every industry, including fintech, making the sector more customer-centric and technologically relevant. The evolution of digital transformation in the fintech industry has become a business imperative for improving customer experience through the development of new services and products ( Alt et al., 2018). The pandemic has accelerated the adoption of fintech and the sector has experienced one of the most drastic changes in its transformation ( Abdul-Rahim et al., 2022; Modgil et al., 2022; Nugraha et al., 2022).

Islamic fintech provided a solution during the pandemic for digital financial innovation with the ease of transactions, without physical meetings ( Hasan et al., 2023; Karim et al., 2022; Yudhira, 2021). Islamic fintech is a growing sector in Indonesia, which has the largest Muslim population in the world ( Muryanto et al., 2021). The Indonesian Sharia Fintech Association (AFSI) noted that from 2020 to 2021, there was a growth of approximately 130% in the Sharia fintech industry. By 2022, it will increase to 180%. Furthermore, The Global Islamic FinTech 2022 Index lists Indonesia as one of the top five conducive ecosystems for Fintech in the rest of the world, including Malaysia, Saudi Arabia, Indonesia, the United Arab Emirates, and the United Kingdom. The potential for Islamic fintech in Indonesia is huge, and the sector is predicted to reach US$179 billion, with a compound annual growth rate (CAGR) of 17.9% by 2026 ( Hudaefi et al., 2023). Indonesia’s National Sharia Finance Committee has made the growth of Sharia fintech a top national priority ( Potkin, 2020).

The basic concept of Islamic fintech in Indonesia revolves around combining the principles of Islamic finance with modern technological advancements ( Oi, 2023) to provide financial services and solutions that are in line with Islamic teachings ( Cherqaoui, 2022). Islamic fintech, also known as “Fintech Syariah,” aims to offer innovative financial products and services that adhere to the ethical and moral guidelines set forth by Islamic law or Shariah. Operating according to Shariah principles, which prohibits interest ( *riba*), uncertainty ( *gharar*), gambling ( *maysir*), and unethical investment ( *haram*) ( Sugiarto & Disemadi, 2020). This is the difference between the conventional fintech and Sharia fintech. In Indonesia, Islamic fintech in operation must meet the operational and license requirements set by the regulator. In terms of supervision, the Indonesia Financial Services Authority (OJK) is responsible for supervising and regulating fintech activities in Indonesia, including Islamic Fintech. In order to improve supervision and customer protection, OJK has issued several regulations and policies related to fintech, such as OJK Regulation Number 77/POJK.01/2016 on Information Technology-Based Money Lending and Borrowing Services, and OJK Regulation Number 13/POJK.02/2018 on Digital Financial Innovation in the Financial Services Sector. Islamic fintech also refers to the Fatwa of the National Sharia Council of the Indonesian Ulema Council (DSN MUI), such as Fatwa DSN MUI Number 116/DSN-MUI/IX/2017 on Shariah-compliant electronic money, Fatwa DSN MUI Number 117/2017 on Information Technology-Based Financing Services Based on Shariah Principles, and Fatwa DSN MUI Number 140/DSN-MUI/VIII/2021 on Shariah-Compliant Securities Through Technology-Based Fund Management Services. DSN MUI is part of MUI, which is tasked with developing the application of Sharia values in economic activities in general, and the financial sector in particular, including overseeing Sharia compliance in Islamic fintech. DSN MUI’s fatwas and guidance serve as important references for Islamic fintech providers and users to ensure compliance with Shariah principles. The institution’s role in issuing fatwas and overseeing their implementation contributed to the development and advancement of Islamic fintech in Indonesia ( Widyastuti et al., 2020).

The collaboration between DSN-MUI, OJK, and other regulatory bodies is crucial in formulating and implementing these regulations to ensure the proper functioning and oversight of Islamic fintech platforms in so that the benefits of Islamic fintech can be maximized. It is known that Islamic fintech can bring several benefits to Indonesia, including financial access, compliance with Sharia, and economic empowerment. Islamic fintech can help increase financial inclusion for micro, small, and medium enterprises (MSMEs) by providing easier access to finance ( Dwijayanti et al., 2022; Putri & Akbary, 2021; Syarifuddin et al., 2021). Islamic fintech platforms also promote financial inclusion by providing access to Shariah-compliant financial services for underserved populations. Islamic fintech can empower the economy by providing easier access to finance for individuals and businesses, promoting entrepreneurship and increasing financial literacy. Islamic fintech conquers financial crises, such as COVID-19, and achieves SDGs for a sustainable nation ( Alshater et al., 2022).

However, from the convenience and benefits of Islamic fintech in Indonesia, there are still challenges that Islamic fintech has to face. First, inadequate regulations, it’s because there are no regulations that specifically regulate or contain Islamic fintech lending ( Hui et al., 2019; Muryanto et al., 2021; Noor et al., 2022). POJK Number 77/POJK.01/2016 leans more towards conventional fintech, creating confusion and legal uncertainty between Islamic and conventional fintech in Indonesia. Second, complicated permit procedures, fintech registration itself is divided into two, for lending-based fintech or Peer to Peer Lending is under the management of OJK. While those under Bank Indonesia (BI) supervision are those related to the payment system. Indonesian Bank Indonesia and OJK should walk together and make fintech licensing only one door. In addition, the existence of special requirements for sharia-based fintech also requires the existence of Sharia Supervisory Board to ensure sharia compliance which also makes regulations through Fatwa DSN-MUI. This complicated licensing has led to the emergence of many illegal fintechs, as evidence by the blocking of 173 illegal online lending entities by the Indonesian Illegal Financial Activities Eradication Task Force (In Indonesia known as Satuan Tugas Pemberantasan Aktivitas Keuangan Ilegal or Satgas Pasti). Third, consumer protection challenges in Islamic fintech in Indonesia encompass issues related to awareness, transparency, data security, dispute resolution, and risk of misuse ( Sugiarto & Disemadi, 2020). The Indonesian Illegal Financial Activities Eradication Task Force is authorized to conduct consumer protection in Islamic fintech. This task force, which consists of OJK, Bank Indonesia, Ministry of Home Affairs, Ministry of Trade, Ministry of Communication and Information Technology, Ministry of Cooperatives and SMEs, Ministry of Investment/Investment Coordinating Board, National Police, Attorney General’s Office, National Intelligence Agency and Financial Transaction Reporting and Analysis centre (PPATK) and other relevant institutions, strengthens regulatory and supervisory relationships to achieve financial system stability. Authorized to regulate the strengthening of supervisory and regulatory relationships between institutions in the financial sector in order to realize Financial System Stability in this case between the Financial Services Authority, Bank Indonesia, the Deposit Insurance Corporation, and the Ministry of Finance. One of them is through the Financial System Stability Committee in the macroprudential and microprudential supervisory mechanisms in the financial system safety net. Furthermore, the strengthening of institutions authorized as regulators and supervisors of the financial sector is carried out to maintain the stability of the financial sector industry and increase public confidence, this aims to create a safer and more environment for Islamic fintech consumers in Indonesia.

The implication of such cases for legal regulation and industry practice is that they may influence how the Islamic fintech industry is regulated and operated. These cases could be an impetus for regulators to strengthen the rules and regulations related to Islamic fintech to protect consumers and ensure compliance with the Sharia principles. As such, legal cases of Islamic fintech operations in Indonesia have an important impact on the development and regulation of the industry. With a strong role in shaping regulations that support growth and innovation, law plays a key role in shaping the future of Islamic fintech in Indonesia. Thoughtful and progressive laws can help create a conducive environment for Islamic fintech to thrive and contribute to its national ecosystem.

Addressing these consumer protection challenges in Islamic fintech requires a combination of regulatory measures, industry self-regulation, consumer education, and technological protection. Ensuring that consumers are well-informed, have access to dispute resolution mechanisms, and are protected from data breaches and non-compliance with Sharia principles is critical to building trust and confidence in the Islamic fintech sector in Indonesia. Indonesia follows the example of other countries that have fintech industries in terms of regulations and policies. The following table compares the regulations in Indonesia with various countries that have fintech industries, such as Malaysia, Saudi Arabia, the UAE, and the United Kingdom:

**Table 1.** A comparative policies and regulations on Islamic fintech industry.

Aspect	Indonesia	Malaysia	Saudi Arabia	UAE	United Kingdom
Sharia Law Compliance	Regulated by the Indonesian Financial Services Authority (OJK) with input from the National Sharia Council.	Regulated by Bank Negara Malaysia (BNM) and the Shariah Advisory Council.	Regulated by the Saudi Arabian Monetary Authority (SAMA) with guidance from religious scholars.	Regulated by multiple bodies, including the Dubai Islamic Economy Development Centre. Sharia scholars to reviewing the products.	Not directly Sharia-based, but ethical and religious financial services are available.
Practical Implications	Regulatory framework envolving to promote Sharia-compliant fintech, with challenges in ensuring wide option.	Established regulatory framework for Islamic finance and fintech, encouraging innovation while ensuring compliance.	Regulatory framework envolving to accommodate fintech while maintaining Sharia principles. Striking a balance between innovation and compliance	Fintech sandbox and regulatory initiatives promoting Islamic fintech while ensuring adherence to Sharia principles.	Fintech-friendly regulations focused on innovation. Ethical and faith-based finance options also available.
Potential Effects on Industry Growth	Large muslim population provides market potential, but regulatory complexities can hinder rapid growth.	Supportive policies have led to the growth of Islamic fintech startups, making Malaysia a hub for Islamic finance.	Growing interest in fintech aligned with Sharia principles, but more clarity needed for widespread adoption.	UAE aims to be a global Islamic fintech centre, regulatory framework encourages startups in this sector.	Fintech industry is thriving, but Islamic fintech is a niche due the non Muslim majority population.
Innovation vs. Compliance Balance	Working to achieve a balance between innovation and Sharia compliance, ensuring protection is a priority.	Balancing innovation with Sharia compliance has been largely successful, resulting in a vibrant Islamic fintech ecosystem.	Striving to strike the right balance between innovation and strict adherence to Sharia principles.	Focusing on innovation within Sharia boundaries, regulatory sandbox supports experimentation.	Innovation is encouraged with ethical considerations being more broadly integrated rather than Sharia-specific.
Market Maturity	Nascent Islamic fintech sector, room for growth with a large unbaked population and growing digital adoption.	Relatively mature Islamic fintech ecosystem with various startups offering a range of Sharia-compliant services.	Developing Islamic fintech sector, gaining momentum with government support and growing interest.	Rapidly growing Islamic fintech market, particularly in Dubai, driven by supportive regulations and infrastructure.	Advanced fintech market, limited islam fintech due to the non-Muslim majority, but ethical finance is well-represented.

Indonesia can emulate the experience of other countries with Islamic fintech industries mentioned above. In terms of Sharia compliance, Indonesia is very weak because the Sharia Supervisory Board operates under the Indonesia Ulema Council (DSN MUI), which is an independent institution. The DSN is authorized to establish and issue Islamic legal opinions (fatwas) regarding economic and financial activities. However, fatwas are religious opinions, not positive law issued by state institutions. Therefore, fatwas do not have binding legal force in a country, thus there are no sanctions or legal threats for non-compliance. Indonesia should emulate other countries that implement Islamic fintech, such as Malaysia, Saudi Arabia, and the UEA, where these institutions are under state auspices, so fatwas and regulations issued constitute positive law and have legally binding force that must be complied with and enforced. The clear regulatory guidelines and supportive infrastructure can help establish the Indonesia as a global fintech hub and attract investment to foster a conductive environment for startups to thrive.

### A Comprehensive Analysis of Policies and Regulations to Emerging Islamic Fintech in Indonesia

The legal landscape of Islamic fintech in Indonesia is complex and dynamic, and is influenced by broader global trends in financial technology. FinTech, which is an amalgamation of finance and technology, represents a fundamental shift in the provision of financial services. The ongoing digitization of finance has disrupted traditional boundaries within the financial sector, resulting in various policy implications. These implications range from fostering innovation and competition while managing risks to expanding regulatory oversight and ensuring the adaptation of public money to the digital world, while striving for robust cross-border coordination.

The advent of digital transformation has affected virtually every industry, including fintech. It has not only made the sector more customer-centric but has also positioned digital transformation as a business imperative ( Chong, 2021). The COVID-19 pandemic has accelerated the adoption of fintech, resulting in significant changes in the industry’s transformation. In Indonesia, Islamic fintech has emerged as a viable solution during the pandemic, offering digital financial innovation with the convenience of remote transactions. Given Indonesia’s status as the country with the world’s largest Muslim population, the growth in Islamic fintech has been remarkable. The Indonesian Sharia Fintech Association (AFSI) reported a substantial increase in the Sharia fintech industry, with growth rates of 130% from 2020 to 2021, and 180% by 2022. Indonesia is recognized as one of the top five conducive ecosystems for fintech globally, as per The Global Islamic FinTech 2022 Index. Projections indicate that Islamic fintech in Indonesia could reach a market size of US$179 billion with a CAGR of 17.9% by 2026, making it a top national priority for Indonesia’s National Sharia Finance Committee.

The fundamental concept of Islamic fintech in Indonesia revolves around harmonizing Islamic finance principles with modern technology. Islamic fintech, often referred to as “Fintech Syariah,” aims to provide innovative financial products and services in compliance with the ethical and moral guidelines established by Islamic law Shariah. This adherence to the Shariah principles differentiates Islamic fintech from conventional fintech, particularly in its prohibition of interest ( *riba*), uncertainty ( *gharar*), gambling ( *maysir*), and unethical investments ( *haram*).

Regulation and supervision are integral to Islamic fintech’s functioning in Indonesia. The Indonesian Financial Services Authority (OJK) plays a central role in supervising and regulating fintech activities including Islamic fintech. The OJK has issued several regulations and policies to enhance supervision and customer protection, such as OJK Regulation Number 77/POJK.01/2016 and OJK Regulation Number 13/POJK.02/2018. Additionally, Islamic fintech operates within the framework of fatwas issued by the National Sharia Council of the Indonesian Ulema Council (DSN MUI), addressing matters such as Shariah-compliant electronic money, financing services, and securities through technology-based fund management services. The involvement of DSN MUI’s in ensuring Shariah compliance is a critical aspect of Islamic fintech oversight. Collaboration among regulatory bodies, including DSN-MUI and OJK, is pivotal for formulating and implementing effective regulations to maximize the benefits of Islamic fintech. Islamic fintech offers Indonesia a range of advantages, including enhanced financial access, Sharia compliance, and economic empowerment. By facilitating easier access to finance for micro, small, and medium enterprises (MSMEs), promoting financial inclusion, and encouraging entrepreneurship, Islamic fintech contributes to the country’s economic development and resilience during financial crises such as the COVID-19 pandemic ( Muzdalifa and Rahma, 2018).

However, Islamic fintech faces several challenges in Indonesia. These include inadequate regulations specific to Islamic fintech lending; complex permit procedures that divide fintech registration between OJK and Bank Indonesia; and consumer protection issues related to awareness, transparency, data security, dispute resolution, and the risk of misuse. The legal cases and challenges within the Islamic fintech industry can influence regulations and operations. These cases may compel regulators to strengthen the rules and regulations to protect consumers and ensure Shariah compliance. The role of law in shaping regulations supporting growth and innovation cannot be understood. A thoughtful and progressive legal framework is essential for creating a conducive environment for Islamic fintech in Indonesia. Addressing consumer protection challenges requires a multifaceted approach that includes regulatory measures, industry self-regulation, consumer education, and technological safeguards. Ensuring that consumers are well informed, have access to dispute resolution mechanisms, and are protected from data breaches and non-compliance with Shariah principles is vital for building trust and confidence in the Islamic fintech sector in Indonesia.

To further develop Islamic fintech in Indonesia further, the country can draw lessons from other nations with established Islamic fintech industries. This includes continually evolving regulatory frameworks that maintain a commitment to Shariah compliance while accommodating innovation. Clear regulatory guidelines, such as those in the UAE, can attract investment and provide a conducive environment for startups. Indonesia can also learn from Malaysia’s inclusive policies, Saudi Arabia’s efforts to strike a balance between innovation and Shariah adherence, and the UAE’s initiatives to establish itself as a global Islamic fintech center. Additionally, exploring ethical finance options alongside Sharia-compliant offerings, as in the United Kingdom, can cater to diverse customer bases. In conclusion, Islamic fintech in Indonesia is a nexus of finance and technology, with tremendous potential for growth and financial inclusion. Effective regulations, collaboration among regulatory bodies, and a focus on consumer protection are critical for harnessing this potential. Learning from global experiences and adopting best practices can help Indonesia create a conducive legal environment for Islamic fintech, while ensuring compliance with Shariah principles and enhancing consumer rights and protection.

## Conclusion

An examination of Indonesia’s legal landscape for Islamic fintech illuminates a rapidly growing sector that harmonizes digital innovation with Sharia principles. This fusion has yielded a dynamic ecosystem that is responsive to the distinctive requirements of Indonesia’s Muslim population. Collaborative regulatory endeavors involving bodies such as the Indonesian Financial Services Authority (OJK) and the National Sharia Council of the Indonesian Ulema Council (DSN MUI) have fostered an environment conducive to Islamic fintech expansion. This regulatory framework, coupled with the impressive growth rates in the Sharia fintech sector, positions Indonesia as a noteworthy player in the global Islamic fintech stage.

Considering the study’s limitations, such as its regulatory-centric focus and the limited exploration of individual Islamic fintech platforms, future research directions can enhance our understanding. Delving into consumer behaviour and trust dynamics within these platforms, investigating innovative technological solutions for bolstering Sharia compliance, and scrutinizing cross-border collaboration potential are avenues for deeper exploration. Additionally, researching the socioeconomic impacts of Islamic fintech, aligning Sharia principles with sustainability goals, and continually monitoring the evolution of the regulatory framework will yield a more comprehensive understanding of the Islamic fintech landscape, ensuring that it thrives within Indonesia’s evolving financial ecosystem.

## Data Availability

No data are associated with this article.
