# Supplementary material for: Exploring The Legal Landscape of Islamic Fintech in Indonesia: A Comprehensive Analysis of Policies and Regulations
Source: F1000Res. 2024 Jun 28;13:21. Originally published 2024 Jan 8. [Version 2] doi: 10.12688/f1000research.143476.2 (PMC11101920; doi:10.12688/f1000research.143476.2)
Supplement: Peer review history

# Reviewer report: Reviewer response for version 2

**DOI:** 10.5256/f1000research.167467.r296652

**Published:** 2024-07-03

**Authors:** Mohamad Abdun Nasir

**Affiliations:** 1 Universitas Islam Negeri Mataram, Mataram, West Nusa Tenggara, Indonesia

The revised version looks much better than the previous one. The author(s) have properly revised on the basis of the reviewer's comments and suggestion. The final draft is more readable.

Is the work clearly and accurately presented and does it cite the current literature?

Yes

If applicable, is the statistical analysis and its interpretation appropriate?

Not applicable

Are all the source data underlying the results available to ensure full reproducibility?

Yes

Is the study design appropriate and is the work technically sound?

Yes

Are the conclusions drawn adequately supported by the results?

Yes

Are sufficient details of methods and analysis provided to allow replication by others?

Yes

Reviewer Expertise:

Islamic law, Islamic economic, Islamic studies

I confirm that I have read this submission and believe that I have an appropriate level of expertise to confirm that it is of an acceptable scientific standard.

# Reviewer report: Reviewer response for version 1

**DOI:** 10.5256/f1000research.157143.r254333

**Published:** 2024-05-17

**Authors:** Mohamad Abdun Nasir

**Affiliations:** 1 Universitas Islam Negeri Mataram, Mataram, West Nusa Tenggara, Indonesia

This article offers a new perspective on the legal ground for Islamic fintech in Indonesia, showing the dynamic growth of fintech and its harmony with Sharia. This is a very interesting and important finding. It shows how sharia and technology can merge to form economic practices complies with technology and modernity.  

 This paper applies appropriate method and data and the analysis is deep and critical. It also uses sufficient bibliographies, both primary and secondary. 

 The minor problem is concerned with some sentences which are complete. This grammatical error can be fixed easily.

Is the work clearly and accurately presented and does it cite the current literature?

Yes

If applicable, is the statistical analysis and its interpretation appropriate?

Not applicable

Are all the source data underlying the results available to ensure full reproducibility?

Yes

Is the study design appropriate and is the work technically sound?

Yes

Are the conclusions drawn adequately supported by the results?

Yes

Are sufficient details of methods and analysis provided to allow replication by others?

Yes

Reviewer Expertise:

Islamic law, Islamic economic, Islamic studies

I confirm that I have read this submission and believe that I have an appropriate level of expertise to confirm that it is of an acceptable scientific standard.

# Reviewer report: Reviewer response for version 1

**DOI:** 10.5256/f1000research.157143.r254337

**Published:** 2024-05-11

**Authors:** Adnan Opeyemi Salaudeen

**Affiliations:** 1 University of Bolton, Bolton, England, UK

**Summary:** The paper conducts a comprehensive examination of the legal landscape of Islamic fintech in Indonesia, aiming to provide insights into the policy and regulatory framework governing this emerging sector. Employing a normative legal research approach with both statutory and conceptual methods, the study analyses existing policies and regulations related to Islamic fintech. The research draws from primary legal materials, such as legislative documents, and secondary legal materials, including scholarly works, to evaluate recent policy developments and regulatory initiatives. Key findings include the rapid growth of Islamic fintech in Indonesia, driven by factors such as the country's sizeable Muslim population, regulatory efforts led by the Indonesian Financial Services Authority (OJK) and the National Sharia Council of the Indonesian Ulema Council (DSN MUI), and the potential for Islamic fintech to contribute to financial inclusion and economic empowerment. However, the study also identifies challenges, including inadequate regulations, complex permit procedures, and consumer protection issues. The paper concludes by emphasising the need for collaborative regulatory efforts, clarity in regulations, and enhanced consumer protection measures to foster the growth of Islamic fintech in Indonesia.

**General Overview: **The paper is clearly and accurately presented. It provides a well-structured overview of Islamic fintech's legal landscape in Indonesia, logically discussing key concepts, regulatory frameworks, challenges, and potential solutions. The study design appears appropriate, employing a normative legal research approach to analyse policies and regulations governing Islamic fintech in Indonesia. 

**Statistical Analysis and Interpretation:** Statistical analysis is not applicable since the study primarily involves legal research and analysis of policies and regulations. Interpreting legal frameworks and regulatory policies is appropriate within the context of the study objectives.

**Recommendations for Improvement:**

- Provide more in-depth discussions on specific regulatory challenges and potential solutions, supported by empirical evidence or case studies where applicable rather than just one summary paragraph on challenges.
- Consider incorporating perspectives from industry stakeholders or experts to provide additional insights into the practical implications of the regulatory framework for Islamic fintech in Indonesia.

Is the work clearly and accurately presented and does it cite the current literature?

Yes

If applicable, is the statistical analysis and its interpretation appropriate?

Not applicable

Are all the source data underlying the results available to ensure full reproducibility?

Yes

Is the study design appropriate and is the work technically sound?

Yes

Are the conclusions drawn adequately supported by the results?

Yes

Are sufficient details of methods and analysis provided to allow replication by others?

Yes

Reviewer Expertise:

Islamic finance, Islamic Social Financer and Islamic Fintech

I confirm that I have read this submission and believe that I have an appropriate level of expertise to confirm that it is of an acceptable scientific standard, however I have significant reservations, as outlined above.

# Reviewer report: Reviewer response for version 1

**DOI:** 10.5256/f1000research.157143.r254340

**Published:** 2024-03-20

**Authors:** Hazik Mohamed

**Affiliations:** 1 Stellar Consulting Group, Singapore, Singapore

First of all, I think regulations in the Fintech space has been lagging and can not keep up with the rapid changes of innovation, so this is not new (both in conventional and Islamic Fintech).

 Due to the lack of a formal Fintech legal framework in Indonesia, this article has tried to put together what is available. The suggestion of measuring impact, aligning with Maqasid Shariah and the UN SDGs is not new but a necessary one. Perhaps, a follow-up paper could be to propose how this can be done. This would be more useful to Islamic regulators and the Fintech ecosystem at large.

 Also, I wanted to point out that gharar is not accurately "uncertainty". It is more closely related to "ambiguity", or specifically "deceptive ambiguity". That is to be consciously deceiving a counter-party through being vague or deliberately ambiguous. "Uncertainty" is the state of not knowing what cannot be ascertained, like the future or unpredictable events. This belongs in God's realm, we can only try to manage it but cannot eliminate it. This is a misconception in many Islamic Finance articles, textbooks and papers.

 The suggestion to draw lessons from other countries should include the emphasis that these lessons drawn need to be specific to Indonesia's needs and gaps. Also, it is important to import good and relevant lessons while learning from the mistakes of others, and not to repeat them.

Is the work clearly and accurately presented and does it cite the current literature?

Yes

If applicable, is the statistical analysis and its interpretation appropriate?

Not applicable

Are all the source data underlying the results available to ensure full reproducibility?

Yes

Is the study design appropriate and is the work technically sound?

Yes

Are the conclusions drawn adequately supported by the results?

Yes

Are sufficient details of methods and analysis provided to allow replication by others?

Yes

Reviewer Expertise:

fintech, Islamic fintech, innovation, sustainability, Shariah applications

I confirm that I have read this submission and believe that I have an appropriate level of expertise to confirm that it is of an acceptable scientific standard, however I have significant reservations, as outlined above.

# Response

**Published:** 2024-03-28

**Authors:** Ramadhita Ramadhita

**Affiliations:** Shariah, Universitas Islam Negeri Maulana Malik Ibrahim Malang, Malang, East Java, Indonesia

Firstly, thank you for your review, the suggestions will be taken into consideration for our next development research.

 Secondly, for the definition of gharar we have learnt more and we agree that gharar leads to ambiguity rather than uncertainty in the law.

 Third, for the third review we will make it more specific as the following data:

 Indonesia can emulate the experience of other countries with Islamic fintech industries mentioned above. In terms of Sharia compliance, Indonesia is very weak because the Sharia Supervisory Board operates under the Indonesia Ulema Council (DSN MUI), which is an independent institution. The DSN is authorized to establish and issue Islamic legal opinions (fatwas) regarding economic and financial activities. However, fatwas are religious opinions, not positive law issued by state institutions. Therefore, fatwas do not have binding legal force in a country, thus there are no sanctions or legal threats for non-compliance. Indonesia should emulate other countries that implement Islamic fintech, such as Malaysia, Saudi Arabia, and the UEA, where these institutions are under state auspices, so fatwas and regulations issued constitute positive law and have legally binding force that must be complied with and enforced. The clear regulatory guidelines and supportive infrastructure can help establish the Indonesia as a global fintech hub and attract investment to foster a conductive environment for startups to thrive.
